# Evaluation of microtensile bond strength and failure type analysis of two different types of universal dental adhesives. In vitro study

**DOI:** 10.1186/s12903-026-08772-y

**Published:** 2026-07-06

**Authors:** Doaa Gamal Ashour, Aya Gamal Ashour, Randa El Naggar

**Affiliations:** 1 Conservative Dentistry Department, Faculty of Dentistry, New Giza University, Km 22 Cairo-Alex Road, Giza, Egypt; 2 Conservative Dentistry Department, Faculty of Dentistry, King Salman International University, South Sinai, Egypt; 3 Conservative Dentistry Department, Faculty of Dentistry, Ahram Canadian University, Giza, Egypt

**Keywords:** Universal adhesives, scanning electron microscope, failure mode, micro-tensile bond strength

## Abstract

**Background:**

The aim of this research was to assess the microtensile bond strength of the recently released Beautibond Xtreme and investigate its impact on the hybrid layer compared to the Single Bond Universal.

**Methods:**

Sixteen extracted teeth were collected and divided into 2 groups (*n = 8*) according to the type of adhesive used, Single Bond Universal (3 M ESPE) and Beautibond Xtreme (Shofu). All teeth’s apical portions were embedded using auto-polymerizing acrylic resin (Imicryl, Konya, Turkey) in a silicone mold till the level of the cemento-enamel junction. The occlusal surfaces were flattened to the level of medium coronal dentin, leaving a residual thickness of about 2.5 mm. The two bonding agents were applied according to the manufacturer’s instructions. Following that, Shofu’s Beautifil II composite was used; each increment was 2 mm high. In order to create serial resin-dentin beams with an approximate area of 1 mm², the teeth were cut perpendicular to the bonding interface with water cooling. The microtensile bond strength test was done by a universal testing machine (5566 S, Instron, Canton, MA, USA) with a load applied at a crosshead speed of 0.5 mm/minute. The specimens were then examined using a scanning electron microscope (SEM) (QUANTA FEG250 Thermo Fisher Scientific, USA). Finally, the failure mode was determined. µTBS data was examined for normality using the Shapiro-Wilk and Kolmogorov-Smirnov tests. The statistical power of the study was set at 80%, statistical significance was set at *P* ≤ 0.05 with a 95% confidence level, and all tests were two-tailed.

**Results:**

For the microtensile bond strength test, intergroup comparison revealed a statistically significant difference between 3 M and SHOFU (*P* = 0.0121), while for the failure mode, there was no statistically significant difference between both groups in failure mode (*P* = 0.2851).

**Conclusion:**

Within the limitations of this study, 3 M showed higher µTBS than Beautibond Extreme. with no statistically significant difference observed in the overall failure mode following microtensile bond strength testing.

## Background

By establishing a strong and durable interface between the tooth and the resin, dentin bonding aims to stop secondary caries. This is accomplished by micro-mechanical hybridization, in which adhesive monomers polymerize inside the collagen matrix after penetrating the demineralized dentin [1].

By removing minerals from the dentin, the etching process reveals a network of porous collagen that permits the adhesive to penetrate. When these monomers polymerize in situ, they form a hybrid layer that binds to both the collagen and the dentin. By strengthening the bond between the dentin and the resin, this hybrid layer lowers the chance of secondary caries and shields the restoration from bacterial infiltration [1].

A solid resin-dentin bond depends on this adhesive-hybrid layer. By interacting with collagen fibrils, hydrophilic acidic monomers in adhesives facilitate penetration into the dentin and influence the strength of the bond. But over time, the bond may become weaker due to nanoleakage and the removal of unreacted monomers, decreasing its durability [2].

To assess the bond strength and modulus of elasticity of mineralized and demineralized dentin, the microtensile test (µTBS test) was developed. Compared to previous techniques, like conventional shear and bond strength tests, this method offered more versatility [3].

Understanding bonding mechanisms and assessing adhesive performance requires an understanding of adhesive interface failure. Fracture patterns are frequently examined by scanning electron microscopy (SEM). SEM offers a thorough understanding of the bonding mechanisms involved and enables the identification of the failure mode, whether cohesive, adhesive, or mixed [4].

The use of functional resinous monomers, such as 10-methacryloyloxydecyl dihydrogen phosphate (MDP) in universal adhesives, which were introduced in 2011, gave dental bonding a new dimension, which is essential for the creation of nanolayers, improving adherence to the tooth surface, and shielding the hybrid layer from hydrolysis by facilitating chemical bonds with dental tissues [5].

MDP’s capability to intermingle with hydroxyapatite powder (HAP) may be impacted by the existence of 2-hydroxyethyl methacrylate (HEMA). This hydrophilic monomer is well known for improving adhesive systems’ capacity to wet dental substrates. But because of its hydrophilic nature, it is vulnerable to water absorption and hydrolysis [6].

HEMA was eliminated from some formulations, such as Beautibond adhesives, which improve dentin’s moisture tolerance and reduce water absorption, in order to lessen the hydrophilicity of universal adhesives. Nevertheless, this frequently resulted in phase separation, which could be avoided by partially substituting methacrylamide monomers for HEMA. Because of their amphiphilic qualities and reduced water absorption, these monomers preserve adhesive stability [7].

In light of these results, the aim of this research was to assess the immediate microtensile bond strength of the recently released HEMA- free Beautibond Xtreme and investigate its impact on the hybrid layer, specifically following the addition of an acid-resistant silane coupling agent to the single bond universal.

The null hypothesis was that there would be no difference in microtensile bond strength between Single Bond Universal Adhesive and HEMA- free Beautibond Xtreme, particularly after modification with an acid-resistant silane coupling agent.

## Methods

### Restorative materials

Two commercially available bonding agents were employed in this study, and their compositions are provided in Table 1. Single Bond Universal (3 M ESPE) and Beautibond Xtreme (Shofu). Both were covered with Beautifil II composite (Shofu). Table 2 shows the classification of the experimental groups according to the universal adhesive systems utilized.

**Table 1:** Materials composition and provider’s details

Material	Composition	pH	Manufacture	Patch Number
Single-bond universal bonding agent	MDP-phosphate monomer, di-methacrylate resin, HEMA, filler, ethanol, water, initiators, silane, vitre-bond copolymer	Mild (pH ≈ 2.7)	3 M ESPE, St Paul, USA	517,571
Beautibond extreme bonding agent	Bis-GMA, TEGDMA, phosphonic acid monomer, carboxylic acid monomer, acetone, H₂O, silane coupling	Mild (pH ≈ 2.5)	SHOFU Inc Corp., Japan	122,235
Beautifil II Resin composite	Bis-GMA, TEGDMA, UDA, S-PRG filler, MF (Multi-functional) glass filler, Nano filler, photoinitiator		SHOFU Inc Corp., Japan	060987

**Table 2:** Groups and their description

Experimental Group	Description
Group: A	Single Bond Universal (3 M ESPE)
Group: B	Beautibond Xtreme (Shofu)

### Specimens and their preparation

Sixteen human third molars that were not carious were gathered. Every tooth used in this investigation was extracted for purposes irrelevant to this study (e.g., orthodontic treatment, impaction, or prophylactic removal), patients’ consents have been approved and signed by them. The teeth were utilized within two months after extraction, after being scaled clean of debris and tissue remains and preserved in a saline solution [1]. Apical portions of all teeth were embedded using auto-polymerizing acrylic resin (Imicryl, Konya, Turkey) in a silicone mold till the level of the cemento-enamel junction. Based on the type of adhesive, the teeth (*n* = 8) were split into two groups.

For all sectioning procedures, teeth were then mounted in an automated diamond saw (Isomet 4000, Buehler Ltd., Lake Bluff, IL, USA). Using a water-soluble anticorrosive cooling lubricant (Cool 2, Buehler Ltd., Lake Bluff, IL, USA) in a 1:33 lubricant-to-water ratio, the occlusal surfaces were flattened to the level of medium coronal dentin, leaving a residual thickness of about 2.5 mm. To create a consistent dentin smear, the dentin surface was polished for sixty seconds using 600-grit water sandpaper [8]. There was no need for an acid etchant because all adhesives were used in the self-etch method. Thus, all adhesives were rubbed using a microbrush based on the following manufacturer’s instructions.

In Single Bond Universal group, the adhesive was applied and rubbed for 20 s, then gentle air drying took place for 5 s, and lastly, it was light cured for 10 s – according to the manufacturer’s instructions. On the other hand, in the Beautibond Xtreme group, the adhesive was used and rubbed for 20 s, then gentle air drying took place, and finally, it was light-cured for 5 s (1200 mW/cm²) – according to the manufacturer’s instructions. The difference in curing time is mainly related to variations in adhesive composition and photoinitiator systems, which influence polymerization efficiency and degree of conversion.

Following that, Shofu’s Beautifil II composite was used. Each increment was 2 mm high, and the whole composite resin block was completed up to a thickness of 5 mm. Every increment was cured by an LED light curing device for 10 s. (VALO™ Cordless, ULTRADENT) To finish the polymerization, the samples were left in saline for a full day.

### Beam preparation

In order to create serial resin-dentin beams with an approximate area of 1 mm², the teeth were cut perpendicular to the bonding interface under water coolant using a low-rate precise cutting tool and diamond cutting disc. This was carried out because it has been suggested that, in order for the results of µTBS testing to be clinically interpretable, the evaluated dentin standard area’s surface area should fall between 0.8 and 1 mm². This area was determined by averaging three measurements made at equal distances on either side of the surface.

After that, the restored teeth were longitudinally sectioned to create composite-dentin beams. Each beam had adhesive applied at the interface between dentin and composite. Acrylic blocks were secured with mounted teeth using a specially made gripping attachment, which ensured alignment with the cutting direction and allowed for precise perpendicular sectioning with respect to the occlusal surface of restored teeth. Under continuous coolant, a 0.3 mm diamond-coated disc (Buehler, IL, USA) was used. The attachment’s metal housing with a soldered square base and two screws allowed for standardized bucco-lingual and mesio-distal sectioning at 90° angles. Beams with a thickness of 1 ± 0.1 mm were produced after sectioning in both directions and a last horizontal cut at the cemento-enamel junction.

Then, 10 beams per group were randomly selected. Dimensions were verified using a digital caliper (Total Tools, Malaysia), and beams were kept in distilled water at room temperature in closed marked containers for each subgroup [9].

### µTBS test

Each beam was attached to the jig at its ends via cyanoacrylate-based glue (Akfix 705 fast adhesive, Turkey). The glue was situated at least 1 mm away from the adhesive interface, and glue accelerator was used to speed up the glue’s hardening process [9]. The microtensile test was done by a universal testing machine (5566 S, Instron, Canton, MA, USA) with a load applied at a crosshead speed of 0.5 mm/minute. Each specimen was attached to a machine jig, then put through testing till it fractured. Newtons (N) were used to record the force at the moment of fracture. The detected force was then divided by each specimen’s surface area to obtain the µTBS. Every value for µTBS is expressed in megapascals (MPa) [8].

After sectioning, around 12 beams were typically obtained for each tooth. Many beams were lost during specimen preparation as a result of cutting techniques or flaws like early debonding or asymmetrical geometry. Testing was not conducted on beams with non-uniform cross-sectional areas, interfacial defects, or dimensions outside the permissible range of 0.8–1 mm².

Specimens that debonded prior to mechanical testing were noted as pre-testing failures and were not included in the statistical analysis. In the end, ten beams per group were chosen at random and tested, the other remaining beams were eliminated because of flaws, incorrect geometry, or handling loss during preparation.

### Scanning electron microscopic (SEM) observation

**Fig. 1:**
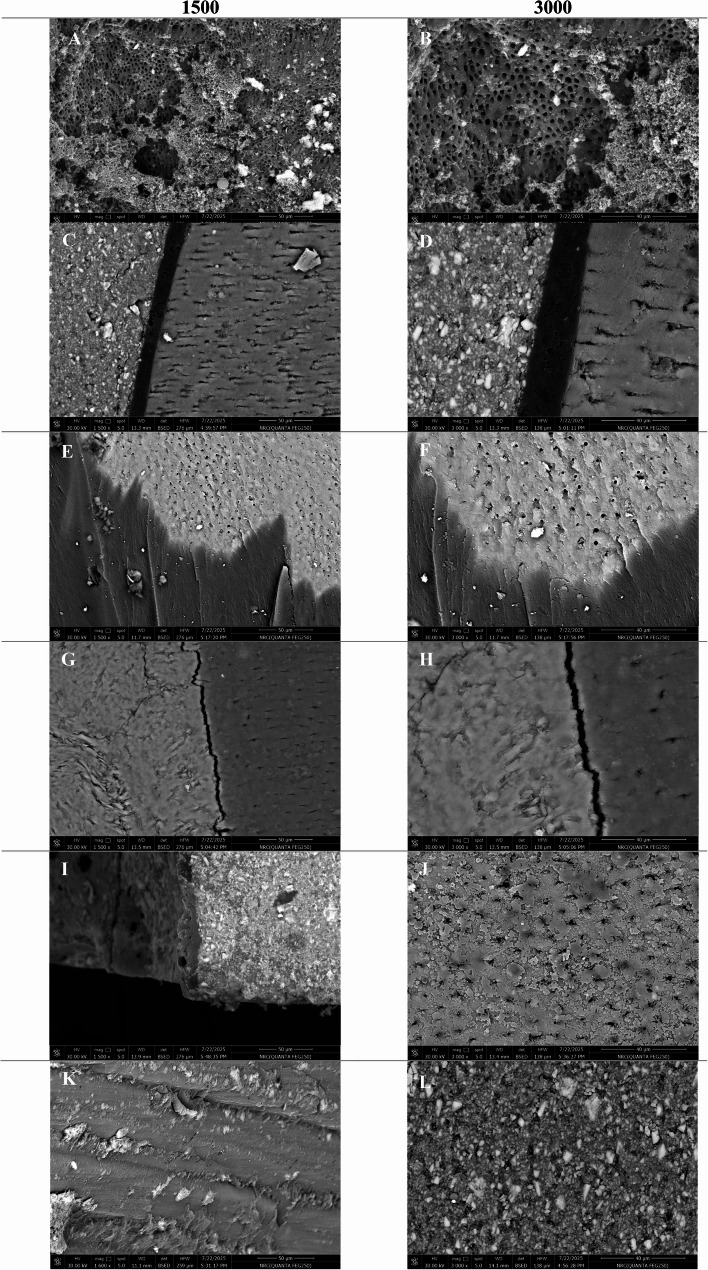
Scanning electron microscope of resin-dentine interface with both magnifications, 1500 and 3000. **A**-**B** SH adhesive failure, **C**-**D** SH cohesive failure, **E**-**F** 3 M mixed failure, **G**-**H** 3 M adhesive failure, **I**-**J** SH mixed failure, **K**-**L** 3 M cohesive failure

**Fig. 2:**
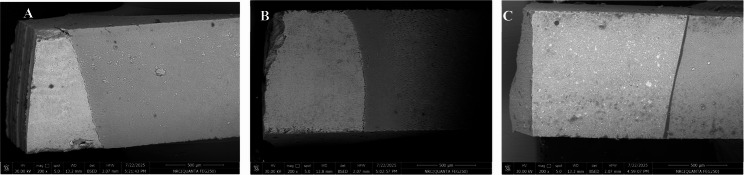
Representative SEM photos with 200 mag. of (**A**) Adhesive failure, (**B**) Mixed failure, and (**C**) Cohesive failure

To assess the bonding surfaces, one sample from each group was prepared for SEM analysis as an illustrative sample for its group, and examined based on fracture patterns. In the central laboratory of NRS (National Research Centre). The specimen sticks’ fractured dentin surfaces were mounted on aluminum stubs and gold-sputter covered with an 18 nm thick layer of gold (80%) and palladium (20%) [1]. The specimens were then examined using a scanning electron microscope (SEM) (QUANTA FEG250, Thermo Fisher Scientific, USA) at 1.2 nm resolution. Images with magnifications of x200, x1500, and x3000 were obtained at 0.2–30 kV, Figs. 1 and 2 [10].

### Failure type analysis

**Fig. 3:**
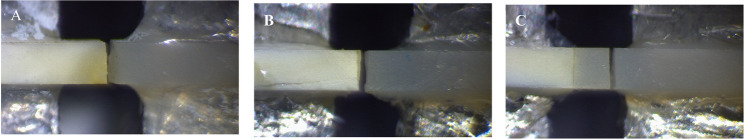
Representative stereo microscope photos at 30x of (**A**) Adhesive failure, (**B**) Mixed failure, and (**C**) Cohesive failure

To determine the mode of failure, each fragmented beam was examined using a Nikon MA 100 stereomicroscope at a magnification of 30X. The failure modes were then categorized in the manner described below: Type I: Adhesive failure between the adhesive resin and dentin; Type II: Mixed failure between adhesive resin and dentin with an adhesive residue on the dentin surface; Type III: Cohesive failure in the resin composite. Stereomicroscopy was used to capture images of the samples’ fracture type, and a computer environment was used to identify the fracture types [1]. Each fracture type’s frequency was expressed as a percentage Fig. 3.

## Statistical analyses

Data was analyzed using MedCalc software, version 22 for Windows (MedCalc Software Ltd, Ostend, Belgium). µTBS data was examined for normality using the Shapiro-Wilk and Kolmogorov-Smirnov tests. The mean and standard deviation (SD) were used to describe the data, which showed a parametric distribution. Intergroup comparison of parametric data was done by an independent t-test, and the effect size was evaluated by Cohen’s d effect size. Frequency and percentage were used to describe categorical data. The chi-square test was used to compare categorical variables between groups. The statistical power of the study was set at 80%, statistical significance was set at *P* ≤ 0.05 with a 95% confidence level, and all tests were two-tailed.

## Results

### Microtensile bond strength (µTBS)

**Fig. 4:**
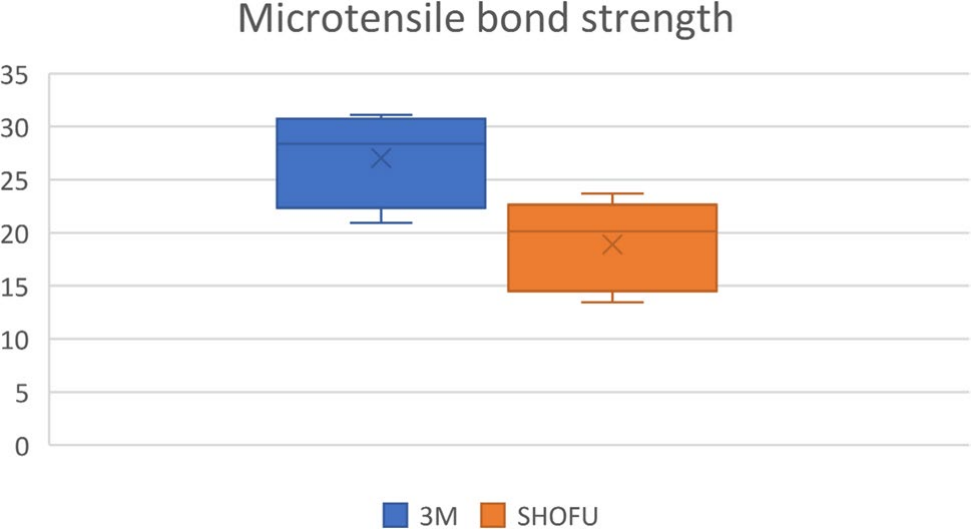
Boxplot of µTBS within both groups

The means and standard deviations of the µTBS, in MPa, were revealed in Table 3, Fig. 4. Intergroup comparison revealed a statistically significant difference between 3 M and SHOFU in µTBS (*P* = 0.0121); 3 M showed 27.03 ± 4.32, and SHOFU showed 18.89 ± 4.26 MPa. The mean difference between them was 8.13 (95% CI 2.25 to 14.02), showing a very large Cohen’s d effect size of 1.92.

**Table 3:** Mean (Standard Deviation) of µTBS values (MPa) of tested groups

Variable	3 M		SHOFU		Difference		
	Mean	SD	Mean	SD	Mean difference	95% CI	*P*-value
µTBS	27.03	4.32	18.89	4.26	8.13	2.25 to 14.02	0.0121*

### Failure mode

**Fig. 5:**
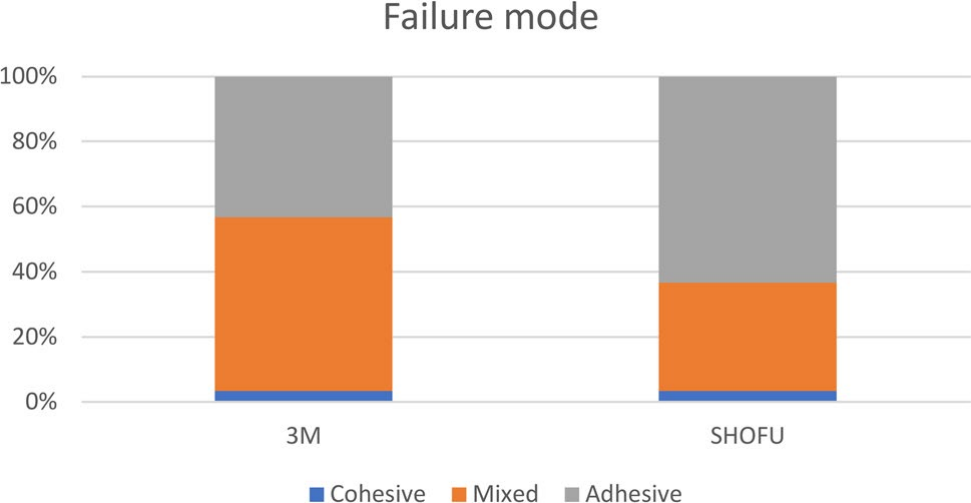
100% stacked column chart showing failure mode distribution within both groups

There was no statistically significant difference between both groups in failure mode (*P* = 0.2851), as shown in Table 4, Fig. 5. In the 3 M group, the most predominant failure mode was mixed (53.3%), followed by adhesive (43.3%), and the least predominant failure mode was cohesive (3.3%). In the SHOFU group, the most predominant failure mode was adhesive (63.3%), followed by mixed (33.3%), and the least predominant failure mode was cohesive (3.3%).

**Table 4:** Failure mode between the tested groups: cohesive, mixed, and adhesive

Group	Failure mode			Row total (RT)
	Cohesive	Mixed	Adhesive	
3M	1	16	13	30 (50.0%)
	3.3% RT	53.3% RT	43.3% RT	
	50.0% CT	61.5% CT	40.6% CT	
SHOFU	1	10	19	30 (50.0%)
	3.3% RT	33.3% RT	63.3% RT	
	50.0% CT	38.5% CT	59.4% CT	
Column total (CT)	2	26	32	60
	3.30%	43.30%	53.30%	
*P*-value	*P* = 0.2851			

### Correlation

There was negligible correlation between µTBS and failure mode within 3 M (rho = -0.169, *P* = 0.7489), while there was moderate negative correlation between µTBS and failure mode within SHOFU (rho = -0.335, *P* = 0.5811), but this was not statistically significant.

## Discussion

Universal adhesives are the result of research efforts to make multistep dental adhesives easier and more applicable. Because of its lower toxicity and adaptability to different adhesive techniques, such as self-etching and etch-and-rinse processes, it has become more and more popular in dentistry.

These universal adhesives’ bonding and efficiency can be significantly impacted by certain ingredients, such as the functional monomer 2-hydroxyethyl methacrylate (HEMA), which dissolves readily in water and other solvents. It is well-known for being hydrophilic and a co-solvent that keeps hydrophobic and hydrophilic monomers in a homogenous state, enhancing adhesive stability and preventing phase separation. Phase separation may occur as the solvent evaporates, particularly in universal adhesives with high water concentrations.

This can jeopardize the bonding in the adhesive-tooth bonding interface. In addition to preventing phase separation, HEMA’s hydrophilic portion can improve wettability and monomer diffusion in dentin due to its polar properties and water solubility. However, because uncured HEMA is hydrophilic, it also reduces the adhesive’s water vapor pressure and makes it harder for it to evaporate when air-dried, which can seriously hinder polymerization. Water sorption in HEMA happens in its uncured and polymerized state due to its high hydrophilicity, which can impair the mechanical properties of the bond by promoting nanoleakage and accelerating bond degradation. Additionally, HEMA is said to interfere with the interactions between hydroxyapatite (HAp) and the phosphate groups of 10-MDP [11].

For this reason, manufacturers are currently advertising HEMA-free adhesives. Similar to Beautibond Xtreme, the goal is to keep the adhesive’s durability and good mechanical qualities. This study compared two universal bonding systems, Single Bond Universal (3 M ESPE), that has HEMA, and HEMA-free Beautibond Xtreme (Shofu), and their impact on microtensile bond strength as well as the failure mode.

In order to standardize the bonding approach, the self-etch technique was used, according to systemic review [12]. In in-vitro studies, it seems that the self-etch adhesion technique has a better choice for fortifying the bond with the dentin. To maximize the universal adhesive bond strength to the enamel, selective enamel etching is recommended when used in conjunction with self-etch adhesion. However, since dentine bonding is the focus of this investigation, a self-etch adhesive technique was employed without any phosphoric acid etching. Because the self-etch adhesive method reduces the likelihood of collagen fibre collapse and the imbalance between how deep the etching goes and how deep the monomer penetrates, it will improve adhesion to dentin and provide better durability by lowering the risk of nano-leakage [13].

Shear bond strength testing is the highest popular method for evaluating bond strength, but some researchers claim that because it only measures force that falls parallel to the material’s surface, its applicability in clinical performance evaluation of dental adhesives is limited due to the pattern of stress distribution. Compared to the microtensile mode, the stress distribution in shear is less uniform. The fact that restorations are typically loaded under a combination of shear and tensile stress rather than in a single mode is another argument against the tensile test [12]. Ultimately, both approaches can provide an indication of the universal adhesive’s bond strength in in-vitro investigations; however, microtensile bond testing is preferred due to its more uniform force distribution, which makes it more applicable and simpler to control and analyze [14, 15].

In this study, intergroup comparison showed a statistically significant difference between single bond universal 3 M and Beautibond Xtreme SHOFU in µTBS, favoring 3 M adhesive. These findings are consistent with [16], who discovered that adhesives without HEMA have a weaker microtensile bond than those with HEMA. These findings were partially explained by the hydrophilic groups used in these experimental adhesives, which are made up of one or two incredibly long chain extenders of ethylene oxide units. A higher network parameter and, consequently, a more flexible polymer can be determined by these long-chain extenders. They also increase the resin blend’s wettability and degree of conversion. A weak resin–dentin interface that is more susceptible to swelling and has high-appearance flaws inside the incompletely infiltrated zones due to the formation of the polymer with high flexibility and high network parameters could be the cause of the low µTBS on dentin and the elevated adhesive failure rates.

According to a different study by Mohammed et al. [17], adhesives with HEMA had stronger bonds than those without. Its hydrophilicity, which improves dentin wetting and thereby strengthens bonds, may be the cause of this. Additionally, HEMA can evaporate from adhesive solutions in very small amounts and has good biocompatibility. Another explanation for this outcome is that HEMA improves bond strength by acting as a wetting agent and assisting monomers in diffusing into the exposed collagen network relatively deeply (3–5 mm) in a clinically manageable amount of time. Wet bonding methods involve filling the spaces between the demineralized dentin collagen fibrils with oral fluids, water, solvent, and/or conditioner. Flow of the resin into any fluid present in the substrate’s gaps and along the collagen fibers is the only method of adhesive resin infiltration. The collagen should ideally be conditioned by the solvent/HEMA combination to stay expanded through adhesive infiltration. HEMA, a major ingredient in several single-bottle marketable dentin adhesives, can significantly lower water evaporation.

Additionally, another investigation by D’Urso et al. [18], discovered that HEMA containing adhesives microtensile bond strength is significantly greater than the HEMA free adhesives microtensile bond strength.

On the other hand, results from [11] show that HEMA has a negative effect on the degree of conversion, which affects the material’s overall mechanical property, but it has no effect on micro-tensile bond strength. He found that, presumably as a result of better wetting qualities, low concentrations of HEMA (10%) raised the 24-hour dentin µTBS. Conversely, over time, reports of universal adhesives with 2.5–10% HEMA content demonstrated stable dentin bonds.

Additionally, another investigation [13] discovered that there was no statistically significant difference in bond strength between HEMA-containing and HEMA-free adhesives. However, HEMA-containing adhesives that contained 10-MDP demonstrated a statistically significant higher bond strength because of the distinct molecular structure of 10-MDP and the hydroxyapatite’s consequent robust and long-lasting calcium adherence.

Even in a long-term bond strength test, Collares et al. [19] stated no difference in bond strength between the HEMA-free and HEMA-containing groups. This may be clarified by HEMA’s low viscosity, which raises the proportion of hydrophobic monomers in the hybrid layer and encourages adhesive resin penetration into the demineralized dentin of the 15% HEMA group.

Finally, a systematic review of Abdelkhalek et al. [20] concluded that the adhesive restorations without HEMA had high retention rates comparable to those with HEMA. Their clinical performance in relation to discoloration and marginal adaptation is still debatable, though.

Under scanning electron microscopy (SEM) in Fig. 1, the mode of failure was investigated in order to correlate µTBS and the mode of failure. The overall failure mode shows no statistically significant difference between the two groups. Although HEMA containing 3 M adhesive demonstrated high bond strength, the most common failure mode in the 3 M group was mixed (involving failure at the dentin/adhesive interface, where part of the adhesive and dentin was damaged), followed by adhesive failure (occurs at the interface between adhesive and dentin). In contrast, the most common failure mode in the SHOFU group was adhesive, followed by mixed, which contrasts with [21], which found the most frequent mode of failure for this bond was mixed, followed by adhesive.

These results can be related to the hydrophilic nature of HEMA. Its presence encourages the creation of a more uniform hybrid layer and improves monomer penetration through the demineralized collagen network. On the other hand, HEMA decreases long-term polymer cross-linking density while simultaneously increasing water sorption. Because of this combination, the interface has sufficient initial micromechanical interlocking but also areas of plasticization and hydrolytic vulnerability, resulting in failure surfaces that contain both cohesive and adhesive components. This is why mixed failures predominate, with purely adhesive failures coming in second.

Conversely, adhesive was the most common failure mode in the HEMA-free adhesive, followed by mixed failures. Lack of HEMA limits monomer infiltration into the collagen matrix and decreases hydrophilicity, which frequently leads to a thinner, less resin-saturated hybrid layer. This increases the possibility of interfacial discontinuities or inadequate encapsulation of the collagen fibrils, which results in weaker immediate interfacial bonding, even though it also decreases water sorption and increases polymer stability. Consequently, debonding mostly takes place at the adhesive–substrate interface, which explains why adhesive failures are more common and mixed failures are less common in this group.

Cohesive failure mode was the least common in both groups Fig. 3. Despite the small correlation between microtensile bond strength and failure mode, the HEMA effect in the HEMA-containing 3 M adhesive is still evident because it reduces the likelihood of adhesive failure compared to the comparator (SHOFU Beautibond Xtreme), where a moderately negative correlation between µTBS and failure mode was found, though it was not statistically significant. The null hypothesis was rejected based on the study’s outcomes.

This study was conducted under controlled in-vitro conditions, which may not fully replicate the oral environment. Aging procedures such as thermocycling were not included, and only two bonding systems were evaluated; therefore, including additional adhesive systems could provide a broader comparison. Furthermore, the SEM analysis was qualitative in nature and relied on visual interpretation, which may introduce subjectivity and does not offer a quantitative evaluation of the interfacial features. Future studies incorporating aging protocols and additional evaluation methods, such as nano leakage analysis, are recommended to further assess the durability of the bonded interface and compare between sialin-containing (Beautibond Xtreem) and other sialin-free bond to investigate the effect of sialin adding to this bond.

## Conclusion

Within the limitations of this study, 3 M showed statistically significant higher µTBS than Beautibond Xtreme. And although there was a difference in the type of failure between each group, it was not considered statistically significant in the overall failure mode results following microtensile bond strength testing.

## Data Availability

All the data made or analyzed during this study are present in this published article.

## Abbreviations

µTBS: Microtensile bond strength
SEM: Scanning electron microscope
MDP: 10-methacryloyloxydecyl dihydrogen phosphate
HEMA: 2-hydroxyethyl methacrylate
HAP: Hydroxyapatite
SH: SHOFU
LED: Light-Emitting Diode curing device
SD: Standard deviation
